# Health Policy and Systems Research Capacities in Ethiopia and Ghana: Findings From a Self-Assessment

**DOI:** 10.9745/GHSP-D-21-00715

**Published:** 2022-09-15

**Authors:** Viroj Tangcharoensathien, Morankar Sudhakar, Zewdie Birhanu, Gelila Abraham, Ayaga Bawah, Pearl Kyei, Adriana Biney, Zubin Cyrus Shroff, Woranan Witthayapipopsakul, Warisa Panichkriangkrai

**Affiliations:** a International Health Policy Program, Ministry of Public Health, Nonthaburi, Thailand.; b Jimma Institute of Health, Jimma University, Jimma, Ethiopia.; c Regional Institute of Population Studies, Accra, Ghana.; d Alliance for Health Policy and Systems Research, World Health Organization, Geneva, Switzerland.

## Abstract

**Introduction:**

Health systems are complex. Policies targeted at health system development may be informed by health policy and systems research (HPSR). This study assesses HPSR capacity to generate evidence and inform policy in Ethiopia and Ghana.

**Methods:**

We used a mixed-methods approach including a self-administered survey at selected HPSR institutes and in-depth interviews of policy makers.

**Results:**

Both countries have limited capacity to generate HPSR evidence, especially in terms of mobilizing adequate funding and retaining a critical number of competent researchers who understand complex policy processes, have the skills to influence policy, and know policy makers’ demands for evidence. Common challenges are limited government research funding, rigidity in executing the research budget, and reliance on donor funding that might not respond to national health priorities. There are no large research programs in either country. The annual number of HPSR projects per research institute in Ethiopia (10 projects) was higher than in Ghana (2.5 projects), Ethiopia has a significantly smaller annual budget for health research. Policy makers in the 2 countries increasingly recognize the importance of evidence-informed policy making, but various challenges remain in building effective interactions with HPSR institutes.

**Conclusion:**

We propose 3 synergistic recommendations to strengthen HPSR capacity in Ethiopia and Ghana. First, strengthen researchers’ capacity and enhance their opportunities to know policy actors; engage with the policy community; and identify and work with policy entrepreneurs, who have attributes, skills, and strategies to achieve a successful policy. Second, deliver policy-relevant research findings in a timely way and embed research into key health programs to guide effective implementation. Third, mobilize local and international funding to strengthen HPSR capacities as well as address challenges with recruiting and retaining a critical number of talented researchers. These recommendations may be applied to other low- and middle-income countries to strengthen HPSR capacities.

## Abstract (precis)

Government investment in strengthening health policy and systems research capacities is needed to enhance the generation of evidence for effective policy making. Researchers’ engagement in the policy-making process helps shape policy-relevant research and support policy-relevant decisions.

## Teaser

**Key Findings:**

- Given the health systems-development challenges in Ethiopia and Ghana, the current level of government investment in health policy and systems research (HPSR) is inadequate to support evidence-informed policies.
- Reliance on donor funding for health policy and systems research is not sustainable, nor does it encourage researchers to be responsive to a country’s health priorities.
- Despite limited HPSR capacity, research portfolios have responded to country priorities in relation to communicable, maternal, neonatal, and nutritional diseases—as well as expansion of health service coverage—as part of moving toward universal health coverage.

**Key Implications:**

- Strengthening and sustaining the capacity of all relevant HPSR institutes are equally important aims that can both be achieved through adequate and sustained investment, opportunities for capacity building among young researchers, favorable incentive schemes, and retention of competent researchers.
- Evidence-informed policy decisions can be enhanced when policy makers are engaged in articulating policy and research questions.
- Researchers’ understanding of the policy process, policy actors, policy entrepreneurs who are key drivers in the policy circle, and current up-to-date discourses in the policy circle facilitates the generation of policy-relevant evidence.

## INTRODUCTION

Through interdisciplinary approaches, the field of health policy and systems research (HPSR) seeks to understand and improve how societies organize themselves in achieving collective health goals, as well as how different actors interact in the policy and implementation processes to contribute to policy outcomes.^1^ Recently, to advance evidence generated from HPSR into program implementation, “embedded implementation research” has been introduced in various settings. Embedded implementation research bridges the gap between researchers and program implementers, not only addressing “what to do given the evidence” but also “how to translate evidence into program implementation and evaluation of outcomes,” identify problems, and propose tangible solutions.^2^ Embedding research, conducted in partnership with policy makers and implementers,^3^ enhances the relevance and applicability of research. Promoting decision makers’ engagement increases the likelihood that they will use research evidence in policy making and health systems strengthening.^4^

The policy analysis triangle^5^ consists of content, context, and process where the outcome of policy decisions is shaped by the interactions among different policy actors. Where agenda-setting is mostly influenced by political decisions, HPSR has played an increasing role in formulating policy and supporting decision making over the past 2 decades.^6^ Policy makers are increasingly demanding evidence to support their decisions and demonstrate their accountability,^6^ which may be due to greater social and political pressure for improved access to services. HPSR has developed in parallel with the growing interest in evidence-informed policy and implementation science; it has also served to underscore the relevance and utility of evidence to policy makers and decision makers.^6^

HPSR evidence—informed by the country’s socioeconomic, cultural, political, and health systems context—is important for objective policy decisions.^7^ This is because HPSR takes into account the complexity of health systems and can support health system strengthening efforts toward the achievement of health-related Sustainable Development Goals (SDGs) by 2030.^8^ The HPSR principles of efficiency and equity are particularly relevant to the low- and middle-income country (LMIC) context. Further, HPSR could have a potential role in informing timely responses to coronavirus disease (COVID-19) by generating evidence on the effectiveness of interventions, disproportionate impacts on vulnerable populations, science to be used in policy decisions, and how health systems can recover.^9^

The translation of evidence into policy is influenced by several factors, notably policy makers’ attitudes toward using evidence, political concerns, and trust in researchers. There is no linear relationship between evidence and policy decisions. Decisions are often value driven and political, not just “evidence-based” choices.^10^ Decisions are often the result of the way research and policy interact with each other in real-life contexts.^11^ Coproduction between researchers and health systems decision makers has been advocated. There are 2 principles guiding successful coproduction: first, researchers and decision makers sharing power by valuing and recognizing their different perspectives and experiences; second, building and sustaining trusting relationships between researchers and decision makers.^12^

For a country to ensure that its health policies are at least based on evidence, it needs 2 things: the capacity to generate policy-relevant evidence and the capacity for this evidence to be translated into policy decisions. Both of these steps require long-term investment to develop, strengthen, and sustain competencies among both individual researchers and research institutes.^13^ They also require long-term involvement of researchers in the policy process so that they understand the complexity of this process and are trusted by policy makers.^14^

Capacity is defined as “the ability of individuals, institutions, and societies to perform functions, solve problems, and set and achieve objectives in a sustainable manner.”^15^ HPSR capacity includes individual, organizational, and societal efforts that support the generation of evidence, which are context-specific for each locality or country and guide policy formulation/reformulation and assessment of policy outcomes and ensure its use for policy. Organizational capacity includes governance and leadership, resources (including human resources, infrastructure, and finance); communication and networking; and technical research capacity.^16^

Significant efforts have been made to improve the capacity to generate evidence that is policy relevant and supports its uptake in LMICs. These include programs that build the capacities of researchers and decision makers, as well as those that enhance the frequency and quality of engagement between these groups.^17^ Indeed, HPSR publications with a lead author from LMICs have increased at a greater rate than those from the biomedical sciences, which is likely due to increased HPSR capacity in LMICs.^18^

Despite these efforts, evidence shows a lower HPSR capacity among researchers from LMICs than among those from upper-middle-income countries. Capacity in upper-middle-income countries is often on par with high-income countries.^19^ In LMICs, donors often represent a large proportion of funding for HPSR, and their funding interests may not always be consistent with national health priorities.^20^ One study shows that donor funding for HPSR-related activities has increased between 2000 and 2014; however, the sources of donor support are skewed, as 93% of HPSR funding is contributed by 10 agencies.^21^ At the same time, HPSR funding accounted for 2% of all donor funding for health and population. Further, small and uncoordinated grants between funders do not address the need for comprehensive capacity development. Lack of national HPSR capacity is a key constraint to health system development as a whole.^22^

The population of Ethiopia is 112.1 million people, 3 times more than that of Ghana. Ethiopia and Ghana have achieved similar levels of life expectancy at birth—66.6 and 64.1 years, respectively. Ethiopia is a low-income country having a gross domestic product (GDP) per capita of US$855.80 in 2019; Ghana is a lower middle-income country with a higher GDP per capita of US$2,202.10. Both countries spent around 3.5% of their GDPs on health in 2018. However, Ghana spent US$77.9, 3 times per capita higher than Ethiopia’s US$24.20. Government spending on health as a percentage of general government expenditure is small, 4.8% in Ethiopia and 6.4% in Ghana—much lower than the Abuja commitment to spend 15% of the general government expenditure on health.^23^ The Table provides a snapshot of country profiles including key health-related indicators in Ethiopia and Ghana.^24^^–^^26^ HPSR funding is important for building HPSR capacity; however, HPSR funding is low in Ethiopia and Ghana. As in other LMICs, research and development expenditure (in all sectors, not only health) in both countries is less than 0.5% of GDP.^27^ The majority of HPSR-specific funding is supported by donors. One study shows donor funding for HPSR-related activities has increased between 2000 and 2014; however, this funding represents 2% of total donor support to health and population programs.

**TABLE.** Key Health-Related Indicators in Ethiopia and Ghana

**Key Indicators, Year** ^a^	**Ethiopia**	**Ghana**
Population (millions), 2019^24^	112.1	30.4
Life expectancy at birth, total (years), 2019^24^	66.6	64.1
World Bank income group category, 2021^24^	Low-income	Lower-middle income
GDP per capita (current US$), 2019^24^	855.8	2,202.1
Research and development expenditure as percentage (%) of GDP; 2017, 2010, respectively^24^	0.27	0.38
Current health expenditure as percentage (%) of GDP, 2018^24^	3.3	3.54
Current health expenditure per capita (US$), 2018^24^	24.2	77.9
Domestic general government health expenditure as percentage (%) of general government expenditure, 2018^24^	4.8	6.4
Domestic general government health expenditure per capita (US$), 2018^24^	5.7	30.3
Out-of-pocket expenditure as percentage (%) of current health expenditure, 2018^24^	35.9	37.7
UHC index of service coverage, 2017^25^	39	47
Proportion of population with large household health expenditures (SDG 3.8.2; 10% threshold); 2015, 2012, respectively^25^	4.9	1.11
Proportion of population with large household health expenditures (SDG 3.8.2; 25% threshold); 2015, 2012, respectively^25^	2.0	0.09
Cause of death, by communicable diseases and maternal, prenatal, and nutritional conditions (% of total), 2019^25^	45	45
Death by noncommunicable diseases (% of total), 2019^24^	43	45
Top 5 leading causes of death, 2019^26^	1. Neonatal disorders 2. Diarrheal diseases 3. Lower respiratory infection 4. Tuberculosis 5. Stroke	1. Malaria 2. Neonatal disorders 3. Stroke 4. HIV/AIDS 5. Lower respiratory infection
Top 5 risk factors that drive the most death and disability combined, 2019^26^	1. Malnutrition 2. Air pollution 3. Water, sanitation, and hygiene 4. Unsafe sex 5. High blood pressure	1. Malnutrition 2. Air pollution 3. Unsafe sex 4. High blood pressure 5. High body-mass index

Abbreviations: GDP, gross domestic product; SGD, Sustainable Development Goal; UHC, universal health coverage.

a Year of most recent data available.

HPSR can help to inform efforts to overcome key health challenges in Ethiopia and Ghana. These include health financing challenges, a high communicable disease burden, and maternal and child health conditions. HPSR may also help to inform strategies to address key risk factors for death and disability related to malnutrition, air pollution, and behavioral factors. Regarding health financing, both countries have high out-of-pocket health expenditure—35.9% and 37.7% of the current health expenditure in Ethiopia and Ghana, respectively. In Ghana, insured populations had high levels of out-of-pocket health spending, largely attributed to the unavailability of drugs at health facilities.^28^ In Ethiopia, around 2.1% of households faced catastrophic health expenditure using the benchmark of a 10% threshold of total consumption.^29^

Both Ghana and Ethiopia have a good record of health development, notably through improved extension of health service coverage to the population. In Ethiopia, universal health coverage (UHC) effective coverage increased from 16% in 2000 to 31% in 2010 and 38% in 2019. Likewise, Ghana’s UHC effective coverage increased from 24% to 34% and to 45% during the same period.^30^ It is therefore interesting to assess whether HPSR contributes to health policy and systems development in these countries.

Reviews of relevant literature have indicated the importance of understanding the policy actors, policy process, and context in which the policy is introduced and discussed. Research evidence is one among several diverse inputs into the decision-making process. Further, policy entrepreneurs—who are strategically located in the policy circle, understand actors, and can influence a successful policy—play an important role.^31^ Given the health challenges Ethiopia and Ghana face (as described), our study seeks to assess each country’s capacity along 2 dimensions: the capacity to generate relevant HPSR evidence and the capacity to inform policy. We also seek to provide policy recommendations to strengthen capacities to both generate and use evidence.

## METHODS

### Data Collection

We applied a mixed-methods approach to our assessment of country capacity. We gathered information through a self-administered survey for several selected HPSR institutes and in-depth interviews with policy makers who are users of research evidence. Data collection was conducted during the first quarter of 2020 when the COVID-19 pandemic emerged.

### Quantitative Surveys

All HPSR institutes in both countries were domestic organizations that have a mandate to generate HPSR evidence and were active in conducting research during the last 3 years. Both public and private institutes were included. Institutes that contracted out HPSR projects to other agencies or researchers were excluded. Individual researchers who conduct HPSR were also excluded, as were agencies that did not have a mandate to conduct HPSR.

In Ethiopia, we mapped and listed 20 national and regional research institutes (universities, private firms, and professional associations). Using expert knowledge and review of institutional websites, documents including gray literature, and publication records of these institutes, we determined that 8 institutes met the inclusion criteria, of which 6 are research units in universities and 2 are private firms. There was no HPSR unit in the Ministry of Health (MOH). All 8 institutes were invited and consented to participate in the self-administered survey.

In Ghana, the HPSR producer terrain was mapped by snowball sampling, expert knowledge, and author identification through HPSR outputs including publications and gray literature during the previous 3 years. Of the 15 HPSR institutes that met the inclusion criteria and were invited to participate, 11 institutes consented, a response rate of 73%. The 11 institutes included 3 health research centers, 5 research units in public universities, 1 national research institute, and 2 nongovernmental organizations.

Based on the reviews of capacity related to HPSR^14^^,^^19^ and experiences gained from establishing and sustaining an HPSR institute during the last 2 decades, we developed, tested, and finalized a questionnaire to assess institutional capacity (Supplement 1). Key modules of the questionnaire include (1) governance and institutional arrangements; (2) HPSR activities; (3) HPSR capacity, including staff and related matters, as well as budget size and sources; (4) prioritizing HPSR; and (5) outputs and dissemination.

To assess HPSR capacity, we gathered data on: (1) number of researchers, their disciplines, recruitment, retention, and capacity development; (2) 3-year trend of size and sources of research funding; (3) research portfolio alignment with country health policy and systems needs; (4) outputs as measured by the number of publications and policy disseminations; and (5) ability to respond quickly to country policy demands.

After the institutions completed the questionnaires, we conducted short interviews with key informants (an experienced senior researcher or manager) from each of the selected research institutes to verify the accuracy of questionnaire responses (Supplement 2). The interviews were conducted from March to June 2020.

### In-Depth Interviews of Policy Makers

Policy makers who use research evidence were identified from government departments or legislative bodies responsible for making health policy decisions at the national or subnational level. Potential interviewees were also proposed by HPSR institutes, which suggested policy makers who use their work.

In Ethiopia, a total of 24 key informants (20 men and 4 women) who are policy makers (4 from the federal MOH, 5 from regional health bureaus, and 15 from federal MOH-affiliated partners) were invited to and agreed to be interviewed. After informants gave their informed verbal consent, we interviewed them in person.

In Ghana, researchers identified 26 policy makers from 11 institutions such as the parliament, government agencies, international organizations, and nongovernmental organizations in health. Of the 12 policy makers (10 men and 2 women) from 6 institutions who consented to be interviewed, 11 were interviewed in person and 1 over the phone interview due to the coronavirus disease (COVID-19) pandemic. After the interviewees gave written consent (except verbal consent for the phone interview), each interview was tape recorded, transcribed verbatim, and analyzed.

We interviewed policy makers on 2 main dimensions during the interviews. First, we looked at their demand for evidence. Specific domains included the frequency and situations in which research evidence was used for policy formulation, the trend and culture of using evidence in policy making, and the most suitable format of research products to promote policy uptake.

Second, we asked interviewees about their experiences working with HPSR institutes. This included the adequacy of research institutes in fulfilling their demand for evidence and key barriers in getting evidence to policy, such as research quality, trustworthiness of researchers (as measured by their competency in producing quality research outputs), policy relevance, practicality and feasibility of policy recommendations made by researchers, and timeliness of research delivery.

### Data Management and Analysis

Interviews were transcribed verbatim and translated to English from the original language where needed. Different codebooks were used for Ethiopia and Ghana, but analysis was guided by 2 broad themes: (1) HPSR institutional governance and capacities as measured by researchers, funding, research portfolios, and outputs; and (2) the relationship between research institutes and policy makers in getting evidence to policy.

In Ethiopia, the lead researcher applied Atlas.ti 7.1.4 to analyze the interview transcripts with 2 outcomes: (1) the codes which were guided by the 2 major themes and subthemes of this study (institutional governance and capacities and relationship with policy makers in informing policy) and (2) new codes emerged from the analysis outside the frameworks. Two other co-investigators, who were assistant coders, reviewed and verified their coding with the initial code by the lead author and after discussions, the final codes were reconciled among the 3 researchers. Results were organized by subthemes and themes and elaborated with quotes.

In Ghana, 3 researchers who were well versed in qualitative data collection and analysis analyzed the qualitative data. The lead researcher provided initial codes to all 24 transcriptions using Atlas.ti 7.5.1. Two PhD students, who were also interviewers, each analyzed 12 transcripts to verify their codes against the initial codes provided by the lead researcher. This checking process, which involved them reviewing and matching codes on a spreadsheet, produced a few additional codes. Using thematic analysis, the codes were grouped into relevant subthemes under the 2 major themes provided in advance: HPSR institutional capacity and getting evidence to policy. Quotes were produced to support the findings.

In both countries, the semistructured interview transcripts were analyzed using Atlas.ti (though different versions), while triangulation was done by comparing them with relevant secondary data. Quantitative survey data analysis was done using SPSS and Excel.

### Ethics Approval

Research ethical clearance was approved by the Ethics Review Committee of the Ghana Health Service (ERC-GHS); Protocol ID number: GHS-ERC 012/11/19; and by the Institutional Review Board of Jimma University, Institute of Health, Ethiopia (Ref. No. IHRPG1/442/2019).

## RESULTS

The research teams in both countries conducted in-depth interviews with key informants during the first quarter of 2020, when the COVID-19 pandemic emerged; however, the pandemic did not interrupt the fieldwork. Online interviews were conducted when in-person data collection was not feasible.

### Governance

In Ethiopia, 6 of the 8 institutes included in our sample are affiliated with government universities overseen by the Ministry of Science and Higher Education. The other 2 are private institutes affiliated with the MOH. All these institutes conduct other health research as well as HPSR. They provide short courses, trainings, workshops, and other formal courses.

In Ghana, our sample included a total of 11 HPSR institutes, 9 public and 2 private nonprofit institutes. Of these 11, 5 are university-based, 5 are affiliated with either the MOH or the Ghana Health Service, and 1 is a national research organization. These institutes conduct HPSR as well as other health research and have an average of 20 years’ HPSR experience. All research institutes have ethics clearance processes within the institute. One institute uses the Ghana Health Service Ethics Review Committee for research ethics approval.

### Institutional Capacities

#### Researchers: Recruitment, Capacity Building, and Retention

In Ethiopia, on average, research institutes have 19 senior researchers (range, 3–46); 55 researchers (range, 3–137); and 23 research assistants (range, 2–80). One research institute is affiliated with a university and has the largest number of researchers. Other institutes have smaller numbers of researchers and lack multidisciplinary work. The majority of researchers working at these institutes are young and thus have few years of work experience. Of the total 561 researchers in all institutes in Ethiopia, 87 (16%) were PhDs, 312 (56%) had Master’s degrees, and 162 (29%) had Bachelor’s degrees.

Recruitment and retention of talented researchers in Ethiopia are challenging, and there are no coordinated strategies to address these issues. This results in most of the experienced and skilled researchers leaving the organization. Replacing these researchers is often difficult.

*The existing environment is unsupportive. This includes limited government funding; absence of capacity building, lack of learning opportunities, low incentives to researchers and lack of mentorship.* —Researcher, Ethiopia

Ghana reports fewer researchers than Ethiopia. Each HPSR institute in Ghana has an average of 9 senior researchers (range, 1–31); 8 researchers (range, 1–15); and 8 research assistants (range, 2–27). Researchers with a PhD consist of 28% of the overall research workforce. Similar to Ethiopia, Ghana faced difficulties in recruiting multidisciplinary researchers. Short courses, internships, secondments, and fellowships are common modes used to strengthen the capacity of young researchers. Of the 11 institutes in our sample, 4 reported that they do not have challenges in recruiting researchers, 5 reported manageable challenges, and 2 reported serious challenges in recruiting researchers. The top 3 barriers to researcher recruitment are insufficient funding, uncompetitive salaries, and unattractive career paths. When asked why researchers leave their jobs in these institutes, respondents’ reasons included resignation for continued study, lack of continued research funding, and uncompetitive salary.

#### Research Funding

In the Ethiopian context, there is no dedicated budget for HPSR from national or international sources; rather, it is considered part of the health research budget. The average annual budget for all types of research (including HPSR) was small: US$61,540 in 2016; US$75,240 in 2017; and US$62,728 in 2018. Only 3 of 8 research institutes received research grants from the government; and these grants were reported to be small and inadequate.

*… there is a wide gap between health challenges in Ethiopia and funding to support HPSR. Government research budget allocated to universities is almost nil, cannot imagine budget for HPSR. —Researcher*

The 5 remaining institutes received research grants mainly from donors. Budget data from private research institutes were not available.

*We should underscore that lack of funds substantially limited our research activities; our funding mainly comes from international donors.*

In Ghana, annual budget figures are available from 8 institutes. The average annual budget for these institutes was US$1.12 million (range, US$0.3–2.0 million) between 2016 and 2018. The majority of research funding comes from international sources, with no budget allocation from the government. Of the 8 institutes, 4 received government core funding, mostly for staff salaries. There is no separate government funding for research grants for HPSR projects.

In addition, in both countries, very few institutes have the budget to enable full access to global databases for domestic and international peer-reviewed journals, so most research institutes have only limited access. Research institutes in both countries did not keep records on the size and sources of research grants from different donors.

#### Prioritized Research Portfolios

Research institutes in Ethiopia are focused mostly on clinical and biomedical research, including national or subnational surveys. HPSR received little attention. The top 5 research priorities in Ethiopia were related to maternal and child health, health service delivery, communicable disease, noncommunicable disease, and mental health. Other important themes, though less prioritized, include human resources for health, road safety, quality of care, and decentralization.

Similar to Ethiopia, the top 5 research priorities in Ghana were related to maternal and child health, universal health coverage, reproductive health, health care financing, and health service delivery; all these areas need evidence from HPSR and implementation research to guide implementation and performance improvement. Prioritization of research areas was guided by relevant national health programs, the global health agenda, and commitments such as SDGs. The MOH and development partners also influenced the research prioritization processes, while other government departments, academia, and health care providers had minimal involvement in this context.

#### Research Outputs and International Linkages

In Ethiopia, between 2016 and 2018, the average number of HPSR projects per institute was around 10. In the last 3 years, there were 8–15 peer-reviewed international publications per institute—more of which were in international than domestic peer-reviewed journals. The majority of peer-reviewed publications were produced by 2 public research institutes and 4 universities, reflecting an uneven distribution among institutes. All HPSR institutes collaborated domestically and internationally on joint research activities, capacity-building endeavors, and policy advocacy.

Research institutes in Ghana have a smaller number of projects per year than those in Ethiopia. On average, they had 2.5 projects in 2016, 2.4 in 2017, and 2.3 in 2018. Other outputs included international peer-reviewed articles, research reports, and posters or oral presentations. Between 2016 and 2018, the 11 research institutes collectively produced an average of 2 domestic peer-reviewed publications and 4.6 international peer-reviewed publications per institute per year. Of the 11 institutes, 7 had international publication records.

All research institutes in both countries used internal and external peer review as quality control measures of research outputs. They also demonstrated domestic and international collaboration through joint research, publication, and capacity building. Audiences of research dissemination included high-level MOH technical officers, academics, health practitioners, and the general public.

### Policy Makers and Research Institute Relationship

In Ethiopia, key informants revealed that evidence had little role in policy or health program development. Often policy development was influenced by past experience, common sense, expert opinion, suggestions from influential donors, and political considerations. There was reportedly not much improvement or an upward trend in using evidence for decision making.

In Ethiopia, policy makers and HPSR institutes identified the disconnect between these groups as the most common bottleneck for using evidence in decision making. Among policy makers, this disconnect was reflected by the absence of involvement of policy makers and key program managers in articulating policy and research questions, as well as by the lack of positive attitudes toward and recognition of the value of evidence in policy. Among researchers, there was a lack of understanding of the “policy process” and difficulty with packaging policy-relevant solutions in a timely manner. Researchers in both countries are mostly affiliated with universities and do not have opportunities to be closely involved with policy makers to understand the policy context and demand for evidence. They viewed factors such as political pressure and donor influence to be out of the control of research institutes. Gaps in effective interactions between researchers and policy makers were voiced by researchers and policy makers in Ethiopia.

*Policy makers did not have good appetite for evidence uptake, rather tended to rely on experts’ opinion, common sense, intuition or experience and politically motivated decisions and sometimes decision can be made all of a sudden without consulting any piece of evidence.* —Researcher, Ethiopia

*Researchers have yet to understand the political dimensions of decision making. Further, the feasibility of policy recommendations depends on the degree to which policy makers and researchers worked together. —*Policy maker, Ethiopia

*We try to connect all our research activities in collaboration with regional health bureau. But we could not influence their policy and health system. —*Researcher, Ethiopia

Similar bottlenecks were reported in Ghana. Although the culture around the use of evidence in decision making was different in Ethiopia and Ghana, respondents from both countries viewed the engagement of policy makers and researchers from the inception of research projects as a facilitator of the research-to-policy process. Timeliness of research findings was also an issue in Ghana. As research evidence is not readily available and research institutes are not agile enough to provide rapid responses for rapid decisions, parliamentarians hire their own research assistants to collect evidence needed for immediate actions.

Common challenges facing researchers in Ghana are a lack of understanding of the policy process and difficulty packaging their evidence into practical and feasible solutions at the operational level. The disconnect between researchers and policy makers and among national and subnational policy makers and parliamentarians were major barriers to evidence-informed policy in this context.

Health policy and systems researchers’ understanding of the political dimensions of decision making is critical in positioning themselves on what, how, and when research evidence should be introduced.

*Producers (HPSR institutes) do not understand politics and how policies are formulated. They tend to behave in ways that distance them from reality. The lack of trust between government and researchers is a major challenge to evidence-based policymaking. —*Policy maker, Ghana

From in-depth interviews in Ghana, 3 enablers emerge for the use of HPSR evidence in making policies. These are the quality and relevance of research evidence; the ways evidence is packaged, delivered, and communicated to policy makers; and constructive relationships and meaningful engagement between policy makers and researchers or research institutes.

*The relationship and the engagement with the program implementers and policy makers matters. This includes their engagement in proposal development to ensure policy relevance, conducting the research. We also involve key programme implementers and subnational level policy makers. Sharing research findings in key meetings of policy makers is important opportunity for policy consumption. —*Policy maker, Ghana

## DISCUSSION

### Current Capacities in 2 Countries

In Ethiopia and Ghana, overall investment in health is inadequate. The current health spending per capita was US$24.2 in Ethiopia and US$77.9 in Ghana, whereas per capita general government health spending was also low—US$5.7 and US$30.3, respectively. These figures should be viewed in light of the fact that estimates suggest that a minimum of US$91 in general government health spending and US$89 in per capita health spending per year is required in low-income countries and LMICs to achieve SDG3.^32^

Although health research and development expenditure is unknown, the overall research and development expenditure was 0.27% GDP (2017) in Ethiopia and 0.38% GDP (2010) in Ghana. This is against the benchmark of 0.58% of GDP among LMICs.

In both countries, policy makers and research institutes voice that the level of HPSR spending is inadequate. Despite some degree of HPSR capacity in producing policy-relevant evidence and contributing to certain policy decisions, HPSR investment is inadequate to support capacity development of young researchers and annual research grants from the government. Unattractive salaries and unappealing career progression paths are major barriers to recruiting young and talented researchers and retaining senior and experienced researchers.

Despite these limitations, HPSR institutes, notably those affiliated with universities in Ghana and Ethiopia, have established collaboration and international linkages with research partners and academic institutes in high-income countries; such collaborations are beneficial in terms of capacity building, coaching, and training of researchers and provide a good platform for joint research and publications.

As most research institutes in both countries were affiliated with a university, they have clear management structures, ethics review processes, and capable researchers. These researchers are based mostly in universities and are somewhat overloaded with teaching obligations. These institutions have international linkages and the capacity to publish in international peer-reviewed journals.

### What Capacities Are Required?

Two sets of capacities are required by HPSR institutes. First, the capacity to produce policy-relevant research requires engagement of policy makers to articulate their policy questions and concerns. Second, the capacity to inform policy decisions requires an effective interface between policy makers and researchers. Researchers’ understanding of the policy process and specific policy elements that are likely to be effective^33^ enhance the likelihood of policy adoption. Policy makers value researchers’ credibility and trustworthiness,^34^ as well as competence and integrity, which facilitate evidence-based policy decisions. The 6 domains of research capacity proposed by the African Health Initiative include: (1) develop skill and confidence, (2) ensure research is close to practice, (3) support linkages and partnership, (4) ensure appropriate dissemination and impacts, (5) build element of continuity and sustainability, and (6) invest in infrastructure.^35^ Further, capacity building to increase research use and collaboration in evidence-informed policy making among policy makers was proposed.^36^

The process of decision making varies by context, content, and actors^37^^,^^38^ and their attributes such as legitimacy, power, and interest.^39^ Actors can either support or not support the policy under consideration. Political context also matters. In Thailand, the political manifesto and commitment to UHC during the election campaign were cornerstones for the UHC agenda,^40^ providing an example where HPSR evidence contributed significantly to policy formulation and subsequent evaluation.^41^ Certain “high stakes” policies and legislation may trigger political interference by industries and their proxies, such as the legislation of control of marketing breast milk substitutes in Thailand^42^ and the tax on sugary/sweetened beverages in South Africa.^43^ Political decisions also play a critical role. In Ethiopia, prioritizing primary health care and later the launch of community health extension workers in 2004 were largely driven by the ruling party’s political responsiveness to rural interests and the adoption of a “developmental state” strategy. This decision was not very clearly influenced by evidence from HPSR.^44^

These examples demonstrate the importance of health policy and systems researchers’ understanding of the political dimensions of decision making. Our in-depth interviews with both policy makers and researchers in Ethiopia and Ghana show large gaps on the part of researchers and research institutions in the understanding of the political dimensions of decision making, knowledge of policy actors who are supportive and resistant, and efforts to identify and work with policy entrepreneurs. They may also work closely with policy entrepreneurs who are strategically located in a policy network and can reach other actors.^45^ With a clear understanding of the complexity of the policy process in their own countries, health systems and policy researchers can decide how and when evidence is useful and applicable to different actors at different phases of the policy cycle.

### Recommendations

Despite limited HPSR capacities in Ghana and Ethiopia and various associated challenges, opportunities for capacity strengthening arise in both settings. We recommend a few tangible solutions that can be applied in both countries.

First, senior researchers’ capacity to understand the national or subnational level policy-making process should be strengthened. This can be done by building relationships with relevant policy actors, specifically through in-person interactions; involvement in current policy discourse; identification and communication with policy entrepreneur(s); and serving as members of a task force, working group, or relevant national committee. The understanding and skills developed through researchers’ close involvement in the policy-making process—witnessing the policy discourses and knowing the policy actors, all of which shape their research into relevant policy that addresses key policy concerns—prompt researchers on what evidence, packaging, and delivery modes may be most effective and relevant to various key actors and policies under discussion. Embedding research in key health programs, with close involvement between research, policy makers, and program implementers, also facilitates evidence-informed policy decisions, especially at the implementation level.

Second, policy-relevant evidence must be generated in a timely manner to facilitate policy decisions. Numerous international publications and guidelines are readily available in the digital era, such as the Cochrane database of systematic reviews,^46^ PubMed, and WHO guidelines and recommendations.

LMICs face 2 major barriers to strengthening HPSR capacity. First, the current academic publishing ecosystem is not in favor of open access and creates unaffordable paywalls.^47^ Second, the prohibitive article-processing cost constrains the ability of African researchers to publish in high-impact journals.^48^

Generating evidence can apply various models. For example, performing a quick synthesis of available relevant international and local published evidence and convening an associated working group to review policy choices and their consequences provides a timely response to political questions that require immediate action. Rapid reviews—a type of systematic review where processes are streamlined to complete the review in a shortened timeframe for immediate policy use—have recently emerged as a useful approach to provide actionable and relevant evidence in a timely and cost-effective manner.^49^ Institutional credibility and trust between institutes and policy makers are boosted when HPSR institutions are responsive and able to respond to immediate policy questions. It also minimizes the mutual mistrust between policy makers and researchers.^50^

Third, HPSR capacity to mobilize local and international funding, both institutional grants and research funding, must also be strengthened, as funding is required to fix various problems related to recruitment and retention of talented researchers. Experienced senior researchers with an understanding of the policy process can produce policy-relevant recommendations. They also offer a mentoring function for promising young researchers, who will, in time, innovate and grow the field further. Investments in research capacity building now will pay dividends in the future for strengthening HPSR institutional capacity raising the profile of the value-add of HPSR among policy makers.

## Supplementary Material

GHSP-D-21-00715-supplement1.pdf

GHSP-D-21-00715-supplement2.pdf

## Notes

**Peer Reviewed**
