# Supplementary material for: Health Policy and Systems Research Capacities in Ethiopia and Ghana: Findings From a Self-Assessment
Source: Glob Health Sci Pract. 2022 Sep 15;10(Suppl 1):e2100715. doi: 10.9745/GHSP-D-21-00715 (PMC9476481; doi:10.9745/GHSP-D-21-00715)
Supplement: GHSP-D-21-00715-supplement1.pdf [file GHSP-D-21-00715-supplement1.pdf]

Supplement to: Tangcharoensathien V, Sudhakar M, Birhanu Z, et al. Capacities for health policy and systems research in Ethiopia and Ghana: findings from a self-assessment. *Glob Health Sci Pract*. 2022;10(Suppl 1):e2100715. https://doi.org/10.9745/GHSP-D-21-00715

Supplement 1.
Capacity assessment of Health Policy and Systems Research (HPSR)

As the world promotes the use of evidence in policy development process, the country's capacity to generate evidence becomes a crucial factor. In health, one important source of such evidence is Health Policy and Systems Research (HPSR). HPSR refers to the production of new knowledge to improve how societies organize themselves in achieving collective health goals, and how different actors interact in the policy and implementation processes to contribute to policy outcomes. It focuses primarily upon policies, organisations and programmes but not the clinical management of patients or basic biomedical research. Their ultimate objective is to promote the coverage, quality, efficiency and equity of health systems.

The objective of this questionnaire is to assess institutional HPSR capacities to generate evidence and inform health policies of the research institutes

Inclusion criteria

- Domestic organisations, public or private, that have a mandate/role/function to produce HPSR **AND**
- Domestic organisations that conduct in-house HPSR with active HPSR profile during the previous three years

Exclusion criteria

- Organisations that do not conduct in-house HPSR, but only allocate budget or contract out HPSR projects to other organisations or researchers **OR**
- Individual researchers who conduct HPSR but are affiliated to organisations that do not have a mandate/role/function to product HPSR **OR**
- Individual researchers who conduct HPSR on their own behalf.

Sample size and sampling strategy for questionnaire

- If the total number in the country is fewer than 30 organisations, all organisations are to be included.
- If the total number in the country is larger than 30 organisations, at least 30 organisations are to be included with a balanced mix of all types i.e. academia, research institute, research unit, research company) in both public, state enterprise, private for not for profit and private for profit.

The respondents of the questionnaire should be the head or senior researcher/manager with longest years of experience in organization or at least 5 years' experience in this role. Only one questionnaire survey is required from each research institute.

Supplement to: Tangcharoensathien V, Sudhakar M, Birhanu Z, et al. Capacities for health policy and systems research in Ethiopia and Ghana: findings from a self-assessment. *Glob Health Sci Pract*. 2022;10(Suppl 1):e2100715. https://doi.org/10.9745/GHSP-D-21-00715

HPSR capacity assessment: questionnaire

---

1. Respondent information

1.1 Name ……………………………………………………………………………………………………

1.2 Contact information

Telephone/mobile ……………………………………………………………………………………………

E-mail ……………………………………………………………………………………………………

1.3 Position (select one)
- ☐ Management function
- ☐ Research function
- ☐ Both

1.4 Years of service in this organisation ………………………………………………………………………………

1.5 Gender ☐ Male ☐ Female

2. Governance and institutional arrangement

2.1 Basic information

2.1.1 Official name of your organisation ……………………………………………………………………………

2.1.2 Year of establishment ……………………………………………………………………………

2.1.3 Organisation website ……………………………………………………………………………

2.2 Organisation type
- ☐ University
- ☐ Not university, please select the following
  - ☐ Research institute
  - ☐ Research unit
  - ☐ Research company
  - ☐ Others, specify …………………………………………………

2.3 Legal status of your organisation
- ☐ Public
- ☐ State enterprise
- ☐ Private, not for profit
- ☐ Private, for profit

2.4 Functions of your organisation (check all that applies)
- ☐ Conduct HPSR
- ☐ Conduct research other than HPSR
- ☐ Allocate fund to others
- ☐ Training/workshop/short course
- ☐ Formal education
- ☐ Policy advocacy/communication
- ☐ Others, specify……………………………………………………………………………………………

2.5 Number of year of HPSR experience that your organisation has ………………………………… years

2.6 Does your organization have the primary focus on conducting HPSR ?
- ☐ Yes
- ☐ No

2.7 Does your organisation have mechanisms to manage conflicts of interest[1]?
- ☐ Yes
- ☐ No

2.8 How does your organisation manage conflicts of interest? (check all that applies)
- ☐ Disclosure e.g. declare financial & non-financial interests
- ☐ Self-regulation e.g. code of conduc
- ☐ Sanctions
- ☐ Others, please specify ……………………………………………………………

---

[1] Conflicts of interest may be defined as circumstances that create a risk that professional judgments or actions regarding a primary interest will be unduly influenced by a secondary interest

Supplement to: Tangcharoensathien V, Sudhakar M, Birhanu Z, et al. Capacities for health policy and systems research in Ethiopia and Ghana: findings from a self-assessment. *Glob Health Sci Pract*. 2022;10(Suppl 1):e2100715. https://doi.org/10.9745/GHSP-D-21-00715

3. HPSR Activities

Activities	No	Yes, Please describe the level of your organization's focus on these activities by scoring (1-least to 5-most)				
**Does your organisation …**		1	2	3	4	5
3.1 prioritize HPSR research in response to country health and health system needs?						
3.2 conduct policy relevant research?						
3.3 mobilize funding for HPSR?						
3.4 communicate and publish research findings?						
3.5 influence policies, provide policy advice based on evidence?						
3.6 network with other domestic and international partners for mutual support?						
3.7 develop and sustain a critical number of health policy and systems researchers with multi-disciplinary skills?						
3.8 Capacity building for policymakers and health managers in using evidence for policy?						

4. HPSR capacity

4.1 Number of research staff by level of experience

- 4.1.1 Senior researcher …………………………
- 4.1.2 Researcher …………………………
- 4.1.3 Research assistants …………………………

4.2 Number of research staff by level of education

- 4.2.1 Bachelor degree …………………………
- 4.2.2 Master degree …………………………
- 4.2.3 Higher than master degree …………………………

4.3 Which of the following Top FIVE disciplines does your organisation have in terms of number of professional staffs?
(select a total of 5 disciplines for health and non-health combined)

Health professions
- ☐ Medicine
- ☐ Dentistry
- ☐ Pharmacy
- ☐ Nursing
- ☐ Veterinary
- ☐ Public health
- ☐ Others, please specify……………………

Non-health professions
- ☐ Statistics
- ☐ Epidemiology
- ☐ Environment
- ☐ Economics
- ☐ Anthropology
- ☐ Political science
- ☐ Sociology
- ☐ Communication
- ☐ Others, please specify…………………

4.4 How do you describe the severity of challenge in **recruiting researchers** to your organisation?
- ☐ No challenge at all
- ☐ The challenge is still at manageable level
- ☐ The challenge affects the quality and timeliness of work

4.5 Barriers in researcher recruitment (check all that apply)
- ☐ Uncompetitive salary and benefit
- ☐ Uncertainty of position and unattractive career path
- ☐ Insufficient budget to hire staff
- ☐ Reputation of your organisation
- ☐ No suitable applicants available
- ☐ Others, specify ………………………………………………………………

4.6 How do you describe the severity of challenge in **retaining researcher** in your organisation?
- ☐ No challenge at all
- ☐ The challenge is still at manageable level

Supplement to: Tangcharoensathien V, Sudhakar M, Birhanu Z, et al. Capacities for health policy and systems research in Ethiopia and Ghana: findings from a self-assessment. *Glob Health Sci Pract*. 2022;10(Suppl 1):e2100715. https://doi.org/10.9745/GHSP-D-21-00715

☐ The challenge affects the quality and timeliness of work

4.7 The overall turnover rate of your organisation in 2016-2019. If possible, please separate by key categories of technical staff. (Turnover rate = $\frac{\text{total number of resigned staff in the year}}{\frac{\text{Staff in Jan} + \text{Staff in Dec}}{2}}$)

2015 ……………………….. % of total staff
2016 ……………………….. % of total staff
2017 ……………………….. % of total staff

4.8 Barriers in researcher retention (check all that apply)

☐ Uncompetitive salary and benefit
☐ Excessive workload
☐ Unattractive career path
☐ Uncertain research opportunities and/or funding
☐ Unsupportive working environments
☐ Researchers wants to continue further study
☐ Personal reasons
☐ Others, specify………………………………………………………

4.9 Which of the following HPSR capacity development programs for your staff does your organisation support? (check all that apply)

☐ None
☐ Scholarships for formal education
  ☐ Postgraduate certificate
  ☐ Master level
  ☐ Higher than master level
☐ Short-course training/workshops
☐ Internship/secondment/fellowship
☐ Individual mentorship
☐ Others, please specify ……………………………………………………………

4.10 Does your organisation have access to databases in the following? (check all that applies)

4.10.1 Domestic peer-reviewed journals ☐ Full access ☐ Limited access ☐ No
4.10.2 International peer-reviewed journals ☐ Full access ☐ Limited access ☐ No
4.10.3 Statistic databases e.g. National database ☐ Full access ☐ Limited access ☐ No
4.10.4 Others, please specify ………………………….. ☐ Full access ☐ Limited access ☐ No

Please answer questions 4.12-4.14 based on the financial information from the average of 3 latest available years, please specify…………………

4.11 Does your country require ethical clearance for HPSR and does it obstruct your HPSR process?

☐ Not require
☐ Require but ethical clearance **does not** obstruct HPSR process
☐ Require and ethical clearance delays HPSR process

4.12 The total amount of **HPSR budget** in USD (including labour cost) …………………………………..

4.13 The **HPSR budget** of your organisation comes from (check only **one**)

☐ Domestic source only
☐ Domestic > international source
☐ International > domestic source
☐ International source only

4.14 Does your organisation receive **HPSR budget** from the government?

☐ Yes, amount in USD …………………………….. ☐ No

4.15 3-year trend of HPSR funding (2016-2018)

☐ Increasing ☐ Decreasing ☐ Not changed

4.16 Did your institute received core funding which was not linked with HPSR projects/programs?

☐ Yes, X Million USD per year, average for the last three years
☐ No

Supplement to: Tangcharoensathien V, Sudhakar M, Birhanu Z, et al. Capacities for health policy and systems research in Ethiopia and Ghana: findings from a self-assessment. *Glob Health Sci Pract*. 2022;10(Suppl 1):e2100715. https://doi.org/10.9745/GHSP-D-21-00715

4.17 How do you describe the level of financial autonomy[2] in your organisation?
- ☐ Low, <30% of overall budget can be allocated freely to different budget lines
- ☐ Moderate, 30-60% of overall budget can be allocated freely to different budget lines
- ☐ High, >60% of overall budget can be allocated freely to different budget lines

5. Prioritising HPSR

5.1 How does your organisation prioritise HPSR areas? (select up to **THREE**)
- ☐ Follow global agenda
- ☐ Follow national or sub-national research agenda
- ☐ Follow the decision by advisory board/committee/internal judgement
- ☐ Institutional interest/plan
- ☐ Follow the funder's conditions e.g. earmarked fund
- ☐ Follow the applicants' interest
- ☐ Follow the decision by external stakeholders [please go to Question 5.2]
- ☐ Others, please specify……………………………………………………………………………………
- ☐ None

Please answer Question 5.2 only if you check 'Follow the decision by external stakeholders' in 5.1

5.2 Which of the following stakeholder groups have involved in the prioritisation process? (check all that applies)
- ☐ Ministry of Health
- ☐ Health providers
- ☐ Other research institutions
- ☐ Professional councils/associations
- ☐ Civil society
- ☐ Other government agencies
- ☐ Development agencies
- ☐ Academic institutions
- ☐ Non-government organisations
- ☐ Other, specify ……………………………………………

6. HPSR outputs and dissemination

6.1 Please rank Top FIVE HPSR themes conducted by your organisation in the past 3 years
- ☐ Health workforce
- ☐ Health financing
- ☐ Service delivery
- ☐ Access to medicines
- ☐ Health information
- ☐ Governance and leadership
- ☐ Communicable diseases
- ☐ Non-communicable diseases
- ☐ Health Technology assessment
- ☐ Reproductive health
- ☐ Maternal and child health
- ☐ Mental health
- ☐ Ageing population
- ☐ Social determinants of Health
- ☐ Burden of diseases
- ☐ Universal Health Coverage
- ☐ Emergency preparedness
- ☐ Others, please specify……………………………………………

6.2 How many HPSR projects has your organisation started in 2016-2018?

Year	Number of project
2016	
2017	
2018	

6.3 How many HPSR publications have the staff in your organisation produced in 2016-2018?

Year	Number in domestic peer-reviewed journals	Number in international peer-reviewed journals

[2] Financial autonomy refers to the organisation's ability to decide freely on its internal financial affairs. The ability to manage its funds independently enables an institution to set and realise its strategic aims. Research institutes receive significant proportion of their funding from funders. Whether this funding is provided as a line-item budget or a block grant, the extent to which it may be freely allocated to different budget lines and the length of the funding cycle are important aspects of financial autonomy.

2016		
2017		
2018		

6.4 How many research outputs have the staff in your organisation produced in 2016-2018?

Year	Number of outputs					
	**Reports/ background papers**	**Policy briefs/ Policy notes**	**Books**	**Conference proceedings**	**Opinion pieces on local media** (e.g. TV, radio, newspapers, magazines, blogs or website)	**Poster/Oral presentations at national conferences**
2016						
2017						
2018						

6.5 Number of events your organisation has held on health policy related issues per year in 2016-2018

Year	Number of events		
	**Public events***	**Private events including stakeholder meetings****	**Meetings with politicians*****
2016			
2017			
2018			

* public events are open to the interested public with no prior invitation required
** private events are closed door events where attendees are by invitation only
*** meetings with politicians and policy makers are held to discuss a health policy issue which can be public or private events

6.6 Taking all projects together, to what extent did your research outputs meet the following quality dimensions during 2016-2018? (check **one for each row**)

Quality	Percentage of the project met					
	**0**	**< 20**	**21-40**	**41-60**	**61-80**	**81-100**
6.6.1 Timeliness of deliverables submitted						
6.6.2 Completeness of deliverables as proposed						
6.6.3 Appropriateness of research methods e.g. data collection, data analysis						
6.6.4 Reliability and validity of research findings						
6.6.5 Policy relevance						
6.6.6 Feasibility of policy recommendations						

6.7 What quality control of research processes are used? (check all that applies)

☐ None ☐ Internal peer review
☐ External peer review ☐ Consultative meeting
☐ Other, specify ………………………………………………………………………

6.8 Please select all existing research collaborations between your organisation and other research agencies or networks **within country** during 2016-2018 (check all that applies)

☐ Jointly conduct research ☐ Jointly author publications
☐ Jointly provide capacity building ☐ Jointly advocate policy
☐ No collaboration ☐ Others, specify……………………………..

Supplement to: Tangcharoensathien V, Sudhakar M, Birhanu Z, et al. Capacities for health policy and systems research in Ethiopia and Ghana: findings from a self-assessment. *Glob Health Sci Pract*. 2022;10(Suppl 1):e2100715. https://doi.org/10.9745/GHSP-D-21-00715

6.9 Please select all existing research collaborations between your organisation and other research agencies or networks **outside country** during 2016-2018 (check all that applies)

☐ Jointly conduct research ☐ Jointly author publications
☐ Jointly provide capacity building ☐ Jointly advocate policy
☐ No collaboration ☐ Others, specify…………………………...

6.10 Who are the targets of your research dissemination? (check all that applies)

☐ Politicians/Parliament members/Member of assembly
☐ High level technical officers in Ministry of Health
☐ Local Chief Executives ☐ Academia/research networks
☐ Mass media ☐ Civil society
☐ General public ☐ Health practitioners
☐ Other, specify ………………………………………………………………………….

6.11 Does your institution have strong linkage with policy makers?

☐ Yes ☐ No

6.12 What are the Top **THREE** mechanisms that your organisation uses to influence policy?

☐ Formal policy platforms e.g. technical working groups, committee
☐ Informal platforms e.g. direct communication and/or advocacy and policy brokering with policymakers
☐ Research communication e.g. reports, policy notes/brief, presentation, publications
☐ Public media e.g. TV, social media, news
☐ Others, please specify ……………………………………………………………

6.13 How many rapid policy requests which required quick response, were made by policymakers to your organisation during 2016-2018?

2016 ………………… requests 2017 ………………… requests 2018 ………………… requests

6.14 Please describe the level of capacity of your organisation to respond to these rapid policy requests

☐ High > 60% of requests ☐ Moderate 30 to 60% of requests, ☐ Low <30 % of requests

6.15 3-year trend of policy demand for HPSR

☐ Increasing ☐ Decreasing ☐ Not changed

6.16 The culture of using evidence in decision making by policymakers in the past 3 years

☐ Improved ☐ Deteriorated ☐ Not changed

6.17 To what extent do you agree that the following factors are barriers in getting evidence to policy?

**Factors**	**Score (1-fully disagree to 5-fully agree)**				
	1	2	3	4	5
6.17.1 Evidence available is not relevant to policy					
6.17.2 Ineffective communication by researchers					
6.17.3 Limited channel to directly link to policymakers					
6.17.4 Low level of political will to use evidence in policymaking					
6.17.5 Policy recommendations are not feasible					
6.17.6 Limited capacity of policymakers to use the evidence					
