# Supplementary material for: Health Policy and Systems Research Capacities in Ethiopia and Ghana: Findings From a Self-Assessment
Source: Glob Health Sci Pract. 2022 Sep 15;10(Suppl 1):e2100715. doi: 10.9745/GHSP-D-21-00715 (PMC9476481; doi:10.9745/GHSP-D-21-00715)
Supplement: GHSP-D-21-00715-supplement2.pdf [file GHSP-D-21-00715-supplement2.pdf]

Supplement to: Tangcharoensathien V, Sudhakar M, Birhanu Z, et al. Capacities for health policy and systems research in Ethiopia and Ghana: findings from a self-assessment. *Glob Health Sci Pract*. 2022;10(Suppl 1):e2100715. https://doi.org/10.9745/GHSP-D-21-00715

Supplement 2. Capacity assessment of Health Policy and Systems Research (HPSR) Interview Guide

As the world promotes the use of evidence in policy development process, the country's capacity to generate evidence becomes a crucial factor. In health, one important source of such evidence is Health Policy and Systems Research (HPSR). HPSR refers to the production of new knowledge to improve how societies organize themselves in achieving collective health goals, and how different actors interact in the policy and implementation processes to contribute to policy outcomes. It focuses primarily upon policies, organisations and programmes but not the clinical management of patients or basic biomedical research. Their ultimate objective is to promote the coverage, quality, efficiency and equity of health systems.

The objective of this interview is to understand the historical trends of country's demands for and supply of HPSR including the culture of and barriers to the use of evidence in policy decision.

Policy makers who use research evidence were identified from government departments or legislative bodies responsible for making health policy decisions at the national or subnational level. Potential interviewees were also proposed by HPSR institutes, which suggested policy makers who use their work.

HPSR capacity assessment: Interview guideline

---

1. Please briefly introduce your organisations including the contribution to HPSR
    - Missions/mandates
    - Governance including financial autonomy
    - Source of funding esp. for HPSR
2. How does your organisation conduct HPSR?
    - Prioritising research, mechanism, and stakeholders involved
    - Ethical clearance process
    - Conducting HPSR, networking, and collaboration
    - Research quality/quality control
    - Communicate research outputs
    - Capacity building
    - Managing conflicts of interest
3. Please give notable examples of the policies that were informed by your research outputs.
4. How has your organisation developed and sustained capacity for HPSR? What are your strengths and weaknesses? And what factors can speed up your HPSR capacity?
    - Human resource and skill-mix & challenges
    - Adequate funding and its sources
    - Supporting facilities and environments
5. Please describe the trend of HPSR demand and supply, culture of using in policy making process in your country. And What are the key barriers in getting evidence to policy? What should be suggestion to mitigate such barriers?
